# Social Media Consumption and Risk of Addiction Associated with Adolescent Disordered Eating Behaviour: An Observational Analysis

**DOI:** 10.3390/nu17183017

**Published:** 2025-09-21

**Authors:** María Peréz-Jiménez, María del Mar Uclés-Torrente, Gema Esperanza Ruiz-Gamarra, Manuel Vaquero-Álvarez, Isabel Maria Blancas-Sánchez, Pilar Aparicio-Martínez, Manuel Vaquero-Abellán

**Affiliations:** 1 Nursing, Pharmacology and Physiotherapy Department, University of Cordoba, 14004 Cordoba, Spain; n02pejim@uco.es (M.P.-J.); n92uctom@uco.es (M.d.M.U.-T.); en1vaabm@uco.es (M.V.-A.); 2 GE10 Clinical and Epidemiological Research in Primary Care (GICEAP), Maimonides Biomedical Research Institute of Cordoba (IMIBIC), 14004 Cordoba, Spain; gemaesprg@gmail.com; 3 Biomedicine Program, Maimonides Biomedical Research Institute of Cordoba (IMIBIC), University of Cordoba, 14071 Cordoba, Spain; 4 Palma del Río Health Center, Dr. Trujillo Street, Palma del Río, 14700 Cordoba, Spain; 5 Grupo Investigación GC09 Nutrigenomics, Metabolic Syndrome, Instituto Maimónides de Investigación Biomédica de Córdoba (IMIBIC), 14004 Cordoba, Spain; h02vaalm@uco.es (M.V.-Á.); isabelm.blancas.sspa@juntadeandalucia.es (I.M.B.-S.); 6 Faculty of Medicine and Nursing, University of Cordoba, 14071 Cordoba, Spain; 7 Occidente Azahara Healthcare Center Córdoba, 14011 Cordoba, Spain

**Keywords:** eating disorders, social networking, self-concept, ideal body weight, adolescent

## Abstract

Objectives: To examine the association between social media (SM) use and content exposure with the risk of developing eating disorders (EDs) among adolescents. Methods: A descriptive observational study was conducted using a structured questionnaire incorporating validated scales. The instrument assessed quantitative and qualitative variables related to eating habits, SM usage, self-esteem, and body image. The sample comprised students aged 12–17 years from a school in Córdoba, Spain. Results: A total of 115 students participated in the study. Among them, 43.5% were identified as being at risk of developing EDs. Of this subgroup, 54.0% were female, with male gender appearing as a protective factor against ED risk. A significant association was found between increased hours of SM use and higher ED risk, with an odds ratio (OR) of 5.54 (95% confidence interval [CI]: 2.03–14.33). Conclusions: The findings suggest that low self-esteem and negative body image are key factors associated with increased ED risk, and that SM may act as an amplifying influence. Preventive interventions should focus on enhancing self-esteem and fostering critical and conscious engagement with SM among adolescents to mitigate the development of eating disorders.

## 1. Introduction

In the digital era, the interaction between society, individual behaviour, perception, and technology has undergone a significant transformation, serving both as a driver of progress and as a source of emerging challenges. Access to information has become instantaneous through the Internet, websites, applications, and particularly social media (SM), establishing Information and Communication Technologies (ICTs) as a core component of modern education and learning processes. The incorporation of ICTs into educational environments offers clear pedagogical benefits; however, it simultaneously exposes young users to significant risks associated with unregulated and often misleading online content [1].

Among these technologies, SM has become the most widely used means of communication across all age groups. For adolescents in particular, it functions as a central space for socialization, self-expression, and identity development [2]. Recent data show that 95.1% of children aged 10–15 are regular computer users, 97.5% are active Internet users, and 68.7% own a mobile phone [3].

Although SM offers undeniable advantages, its inappropriate or excessive use has been linked to psychosocial and health-related consequences. Particularly troubling is its influence on body image, which may foster dissatisfaction and contribute to the development of EDs [4,5]. The rapid circulation of unverified or misleading content, combined with addictive usage patterns, can subtly but powerfully shape perceptions and behaviours, often beyond users’ conscious awareness.

EDs are recognized as multifactorial conditions influenced by biological, psychological, and social determinants, yet body dissatisfaction consistently emerges as one of the strongest risk factors for disordered eating. Numerous studies confirm that SM accelerates the spread of unrealistic stereotypes and idealized body images [3]. Platforms frequently showcase digitally altered or artificial intelligence-generated content that promotes unattainable standards of thinness and physical “perfection.” Even materials marketed as health-oriented often misrepresent reality, reinforcing dissatisfaction among adolescents. Those with low self-esteem appear particularly vulnerable, as they may come to believe that only these artificial ideals are socially acceptable [6].

Given these trends, researchers have described EDs as a “silent pandemic” [7,8]. Globally, the prevalence of EDs has shown a sustained increase over the past decades. Between 1990 and 2021, the age-standardized global rate rose from approximately 300 to 355 cases per 100,000 inhabitants, reflecting a progressive increase across all age groups [9]. Recent studies further estimate that more than one in five children and adolescents present disordered eating behaviours, even if they do not all meet full diagnostic criteria [10]. In Spain, the overall prevalence of EDs is estimated to range between 1% and 4% of the population, with significantly higher rates reported among adolescent females (4.1–6.4%) compared to males [11]. For instance, in the Community of Madrid, a population-based study found a prevalence of 3.4% of EDs among female secondary school students [12]. These figures are comparable to those reported in other European countries and in the United States, where the prevalence of disordered eating behaviours also exceeds 20% among adolescents [7,10]. Specific platforms play a notable role; for instance, studies on TikTok show that children and adolescents with EDs frequently encounter anorexia- or bulimia-related material, even without actively seeking it [13,14]. Content such as “thinspiration” and “fitspiration” acts as a potent trigger for imitation of disordered eating or self-injurious behaviours [11,12,13,14,15].

These results were also confirmed by the study of Nagata J. et al., whose analysis of 10,092 adolescents aged 11 to 15 years indicated that 69.5% reported having at least one social media account. The most commonly used platforms were TikTok (67.1%), Instagram (66.0%), and YouTube (64.7%). Social media use was also common among participants younger than 13 years, with 63.8% reporting active accounts despite age restrictions. On average, these under-13 users reported 3.38 accounts, with TikTok being the most prevalent (68.2%) and most frequently cited as their primary platform (39.0%) [16]. This constant engagement creates sustained exposure to curated images and messages promoting beauty standards, dietary restrictions, and idealized body types, raising significant concerns for adolescent mental health and eating behaviours [17,18,19].

Community-based studies confirm the complex associations between SM use, body dissatisfaction, and maladaptive practices such as restrictive eating or compulsive exercise. An Australian study involving 681 adolescents (49% female; mean age 12.76 years) revealed significant links between SM exposure, body dissatisfaction, and both disordered eating and muscle-enhancing behaviours [20]. The ubiquity of “influencers,” combined with commercial advertising and peer-driven content, further amplifies the belief that only certain body types are acceptable.

Furthermore, adolescents frequently encounter so-called “fitness influencers” or unqualified individuals offering nutritional and exercise advice that lacks scientific rigour. This dissemination of unverified guidance further reinforces distorted body ideals, particularly those affecting females, although recent trends indicate a growing impact on males as well. The constant portrayal of romanticized bodies in the media, advertising, and social networks intensifies the belief that only certain body types are acceptable, further exacerbating body dissatisfaction and vulnerability to eating disorders among adolescents [21,22].

Given that EDs typically emerge during adolescence and often begin with subtle behavioural or emotional symptoms, educational professionals, caregivers, and health providers must be vigilant. Early indicators, such as restrictive eating, body dissatisfaction, avoidance of social meals, or emerging secretive behaviours, can be critical warning signs if thoughtfully recognized [23,24]. Timely identification of these early markers enables prompt intervention, which is associated with higher recovery rates [18] and is central to modern evidence-based approaches to ED management [24,25]. In light of these findings, the present study aims to investigate the prevalence of EDs among students, raise awareness, and contribute to preventive strategies. Addressing this growing public health concern demands a comprehensive approach, combining research, education, and media literacy to mitigate the detrimental impact of SM on the health and well-being of young people.

## 2. Materials and Methods

### 2.1. Study Design

This research employed an observational, descriptive, cross-sectional design. Data were collected from students belonging to different academic groups in middle school during the academic school year of 2023–2024, with the gathering of data finalized in May 2024. They were invited to participate by completing a structured survey after confirming participation with underage students and their guardians/parents.

### 2.2. Sample Size

A non-random convenience sampling was carried out from a population of students belonging to a school in Córdoba capital, from the 1st to 4th years of Compulsory Secondary Education (known as ESO in Spanish), with ages between 10 and 17 years. The minimum sample size was 77 people, based on expected frequency 5.3% [17] and a precision of 5%, using the EpiInfo 7.0 programme. The final sample was 115 students belonging to the courses mentioned above. Each course was composed of four groups (A, B, C and D), of which only two were taken randomly. The selected courses were as follows: 1st E.S.O (A and C); 2nd E.S.O (B and D); 3rd E.S.O (A and D); 4th E.S.O (A and B). The students from the study were categorized as having a medium-high socioeconomic level and a Caucasic background. Of these, 45.21% (52 students) were boys and 54.78% (63 students) were girls. The mean age was 13.85, with a standard deviation of 1.14.

### 2.3. Variables

The questionnaire was developed after reviewing the existing literature, as no single validated tool was identified that encompassed all the variables of interest for this study. For this reason, several standardized instruments were combined and segmented by test to address each key relevant point to the analysis [26].

In this sense, to evaluate the risk of EDs, diverse standardized self-report screening instruments were evaluated by the authors. The Eating Attitudes Test-26 (EAT-26) was included, as it is one of the most widely used measures for assessing disordered eating behaviours and concerns related to dieting, bulimia, and food preoccupation [27]. This test is a multidimensional instrument with excellent reliability, sensitivity, and specificity, making it ideal for screening possible eating disorders in at-risk populations. Another possible test was the SCOFF questionnaire, a brief five-item tool, employed given its utility as a rapid screening measure for identifying core features of eating disorders, particularly in non-clinical and large-population settings, with a greater focus on bulimia. In addition, another validated instrument was the Spanish version of the Eating Disorder Examination Questionnaire (EDE-Q), a self-report instrument adapted from the Eating Disorder Examination (EDE) interview, designed to assess the key attitudinal and behavioural features of eating disorders. It evaluates four core domains: Restraint, Eating Concern, Shape Concern, and Weight Concern, along with providing a global score that reflects overall eating pathology. Based on the specialization of each instrument, the EAT-26 was selected, since it is one of the border tests, with inclusion of diverse segments and without a specific analysis in EDs. In addition, other tools, while not diagnostic, serve the critical purpose of flagging individuals who may be at risk and in need of more comprehensive clinical evaluation.

Based on the previous analysis, the final instrument included the sociodemographic section, EAT-26, Rosenberg Self-Esteem Scale, Body Shape Questionnaire (BSQ), and Social Media Disorder Scale (SMDS).

Firstly, sociodemographic information was collected, including sex, age, academic year, weight, and height, together with items related to the type of SM and the content most frequently accessed by participants.

The EAT-26 was used to evaluate the risk of EDs [28]. This instrument consists of 26 items rated on a Likert-type scale with five response options: 0 points for “never” and “almost never,” 1 point for “often,” 2 points for “very often,” and 3 points for “always.” Item 25 is reverse scored, with “never” valued at 3 points and “very often”/“always” at 0 points. According to established thresholds, scores < 11 indicate no risk of ED, scores between 11 and 19 indicate moderate risk, and scores ≥ 20 indicate high risk.

Self-esteem was assessed using the Rosenberg Self-Esteem Scale [29], which includes 10 items addressing self-respect and self-acceptance. The scale comprises both positively worded items (1, 2, 4, 6, 7) and negatively worded items (3, 5, 8, 9, 10). Responses are rated on a four-point Likert scale (“strongly agree,” “agree,” “disagree,” “strongly disagree”). For scoring, positive items are assigned values ranging from 4 to 1, while negative items are reverse scored from 1 to 4. Body image perception was measured using the Body Shape Questionnaire (BSQ) in its validated 14-item version [30].

The variable “use of social networks” was evaluated through the SMDS in its short version [31]. While the original scale includes 27 items, the short form consists of 9 items, each corresponding to one of the nine DSM-5 criteria for addictive behaviours related to gaming and SM. Responses are dichotomous (“yes” = 1; “no” = 0). A score ≥ 5 indicates the presence of SMD.

### 2.4. Data Collection Procedure

Data collection was carried out from March to April 2024. For data collection, the following procedure was carried out, which was composed of several phases:

#### 2.4.1. First Phase: Selection of Centre and Corresponding Permits

Once the centre in which the research was going to be carried out was selected, we carried out the following procedure. First, we contacted the management of the said centre to explain the objectives of the project, and to request their consent to carry it out, by means of an email and a subsequent meeting. Once approved, documentation was sent to the ethics committee for approval (Code: 5864). Next, to inform the families of the students, an authorization form was made, explaining what the research consisted of and requesting their consent and agreement for their children to participate in the study.

#### 2.4.2. Second Phase: Information to Students

All participants were informed of the purpose of the study, the voluntary nature of their participation was indicated through the guarantee of anonymity and confidentiality of data, and their consent in addition to that of their parents.

#### 2.4.3. Third Phase: Data Collection Through the Survey

The different surveys selected for the research were presented through Google Forms anonymously online, and physically through printed questionnaires for those students who did not have an electronic device to carry it out. Through the Google Classroom platform, students had access to the survey link so that they could carry it out. For this, they used the electronic devices that are allowed to be used in the centre, in this case, tablets and computers, due to the Andalusian law prohibiting mobile devices in educational centres. The completion time for the survey was around fifteen minutes.

### 2.5. Statistical and Epidemiological Analysis

Descriptive statistics were performed using frequencies and percentages for qualitative variables and means with standard deviations for quantitative variables. The Kolmogorov–Smirnov test was applied to assess the normality of the data. For inferential analysis, odds ratios (OR) with 95% confidence intervals (CI) were calculated to estimate the strength of association between the risk of EDs and the variables of interest. The chi-square test was used to examine relationships between qualitative variables, while analysis of variance (ANOVA) was employed to compare groups for quantitative outcomes.

### 2.6. Ethical Considerations

This research adheres to the professional codes of ethics of the professional profiles working within it, as well as the Declaration of Helsinki and all ethical and legal requirements promoted in our country to ensure both confidentiality and good practices from the point of view of bioethics. In accordance with Organic Law 3/2018 of 5 December, on the protection of personal data and guarantee of digital rights, the data provided by the user will be incorporated into an automated file, which will be processed exclusively for the purpose described.

## 3. Results

Table 1 presents the sociodemographic distribution of the sample (N = 115). Slightly more than half were female (54.8%), with a mean age of 13.85 years (SD = 1.41). Age was categorized into three groups (12–13, 14–15, and 16–17 years). Academic year distribution was relatively balanced, with the largest representation from 3rd ESO (27%). Anthropometric characteristics are shown in Table 2: the mean weight was 53.2 kg and the mean height was 161.4 cm, yielding a mean BMI of 20.0. Most participants (92.2%) were within the healthy BMI range, while 2.6% were underweight and 3.5% were obese.

Regarding weight, in Table 2, the minimum was found to be 42 kg and the maximum 90 kg, with a mean of 53.2 kg. Concerning height, our minimum and maximum range was between 142 cm and 185 cm, with a mean of 161.4 cm. In order to work better with these variables, BMI was calculated according to the WHO BMI tables from 2007 (39). Most of the sample was at a healthy weight, with 106 adolescents (92.2%) and a mean BMI of 20.04.

All participants indicated having a SM account (100%), using Instagram (72%), TikTok (68%), and, in low percentage, YouTube (60%). As shown in Table 3, nearly all participants (96.5%) reported daily SM use (Table 3). Most began between ages 10 and 16, with almost 8% starting before age 10. Daily time spent varied, though nearly half (46.1%) used SM 1–3 h/day, while 26.1% exceeded 3 h/day. Over one-third (36.5%) perceived a negative impact of content. More than half reported using SM for exercise (55.7%) and nutrition (57.4%). Based on the SMDS, 33% of adolescents met the criteria for SMD.

According to Table 4, physical exercise was reported by 87.8% of participants. Motivations varied: 47.8% exercised for health or fun, while more than half cited body dissatisfaction, either as the sole reason (15.7%) or in combination with health/fun (36.5%).

The variables related to self-esteem and body self-perception are shown in Table 5. This table reflects levels of self-esteem and body perception. Nearly half (48.7%) had medium self-esteem, 34.7% had low self-esteem, and only 16.5% had high self-esteem. Body dissatisfaction was widespread, with 50.5% reporting mild to extreme concern.

### 3.1. Risk of ED: Prevalence and Associated Factors

Overall, 45.2% of the sample were at risk of ED (EAT-26 ≥ 11; Table 6). Prevalence was higher among females (53.9%) than males (30.8%). By age, risk was highest in 12–13 years (48.7%), followed by 14–15 years (41.2%), and 16–17 years (37.5%).

Table 7 shows the relationship between the variables included in the study and the likelihood of suffering from an ED. It was observed that the female gender showed an OR = 2.64, 95% CI (1.22–5.69), and a *p*-value of 0.012. Furthermore, the use of social media for more than 3 h daily presented an OR = 5.54, 95% CI (2.03–14.33), and a *p*-value < 0.001. Within the same group, those whose content did not include information on exercise showed an OR = 2.48, 95% CI (1.15–5.35), and a *p*-value of 0.019, while those who did not consume dietary advice, nutrition, etc., compared to those who did, had an OR = 6, 95% CI (2.56–14.07), and a *p*-value < 0.001. These results indicate that there is a positive association between the variables described above and the risk of appearing to have an ED.

In relation to age, belonging to a range between 14 and 15 years presents an OR = 1.17, 95% CI (0.26–5.28), and *p* = 0.705. Having a higher BMI, within the ranges of overweight and obesity, showed an OR = 2.60, 95% CI (0.45–14.9), and a *p*-value of 1.244. Regarding the age of onset of social media use, the group with ages between 12 and 16 years presented an OR = 1.71, 95% CI (0.39–7.60), and *p* = 0.730. Low self-esteem indicates an OR = 9.64, 95% CI (2.73–34.08), and *p* < 0.001, while high body concern indicates an OR = 161.50 and 95% CI (18.23–142.97).

**Figure 1:**
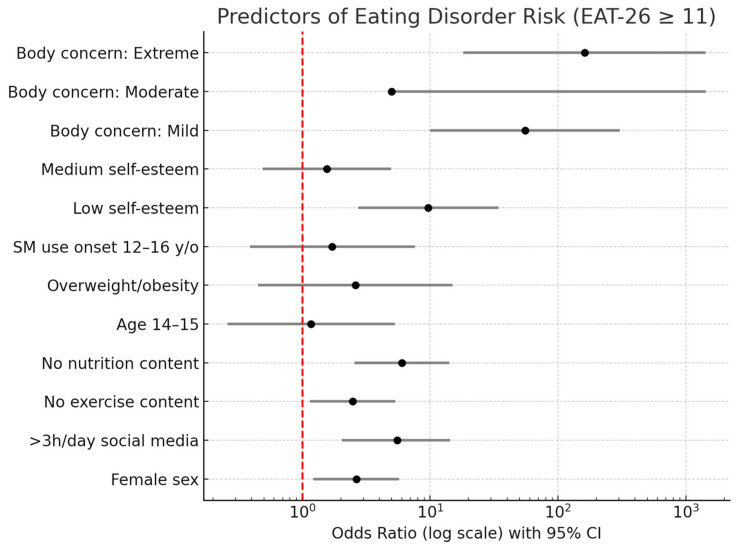
Forest plot of predictors of eating disorder risk (EAT-26 ≥ 11). Predictors expressed as OR on a logarithmic scale with 95% confidence intervals. Black dots represent OR estimates, gray lines show the confidence intervals, and the red dashed vertical line at OR = 1 indicates no association. Predictors to the right of the line (e.g., body concern, low self-esteem, high social media use, female sex, overweight/obesity) increase risk, while those to the left (e.g., medium self-esteem) reduce risk.

Figure 1 showed the ORs with 95% CI for variables associated with ED risk. Strong associations were observed for low self-esteem (OR = 9.64, 95% CI 2.73–34.08), extreme body concern (OR = 161.5, 95% CI 18.23–1430.97), and mild body concern (OR = 55.2, 95% CI 9.97–306.17). In addition, excessive SM use (>3 h/day, OR = 5.54, 95% CI 2.03–14.33) and not seeking nutritional content (OR = 6.00, 95% CI 2.56–14.07) were significantly related to higher ED risk. Female sex also increased risk (OR = 2.64, 95% CI 1.22–5.69). In this sense, females reported a substantially higher prevalence of ED risk (53.9%) compared to males (30.8%). Regarding age, the highest prevalence was observed in the youngest group (12–13 years, 48.7%), followed by those aged 14–15 years (41.2%), and those aged 16–17 years (37.5%). These results suggest that early adolescence, especially among girls, is a critical period for vulnerability to disordered eating behaviours. Beginning SM use before age 10 showed significance for ED (OR = 2.03, 95% CI 0.48–8.59, and a *p*-value of 0.045). These results indicate that there is a negative association between the variables described above and the risk of appearing to have a SMD.

### 3.2. Predictors of SMD and Psychosocial and Eating Behaviour Associations

Table 8 shows the relationship between the variables included in the study and the risk of suffering from a SMD. The analysis identified predictors of problematic SM use. Adolescents spending more than 3 h/day on SM showed the strongest association with SMD (OR = 18.07, 95% CI 5.81–56.20). Additionally, using SM to escape negative feelings (OR = 11.33, 95% CI 3.66–35.09) and failing to reduce usage despite attempts (OR = 5.64, 95% CI 2.33–13.60) significantly increased risk. Early onset of SM use (<10 years) was associated with higher odds, although not statistically significant.

**Figure 2:**
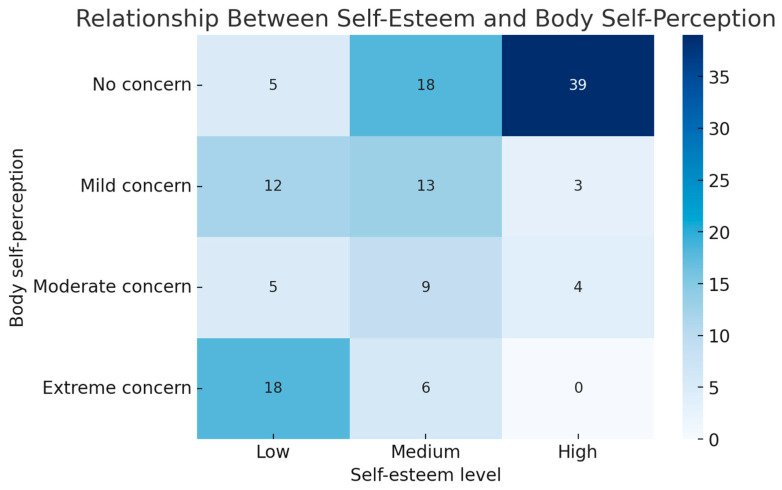
Heatmap of self-esteem level and body perception in relation to the risk of EDs (EAT-26 ≥ 11).

Additionally, the analysis through a heatmap demonstrated a clear pattern linking low self-esteem with moderate to extreme body concern, while high self-esteem was predominantly observed among adolescents reporting no body concern (Figure 2). This strong, statistically significant association (*p* < 0.001) highlights the interplay between body image dissatisfaction and reduced self-esteem, both of which are key risk factors for disordered eating behaviours.

Finally, Table 9 shows the relationship between two other important variables in our study—self-esteem and body perception. A value of *p* < 0.001 is observed, finding significant relationships between these variables. Based on these results, the analysis was adjusted for sex, age, BMI, SM use, and psychosocial variables. Significant predictors of ED risk included female sex (aOR = 2.45, 95% CI 1.12–5.36), >3 h/day SM use (aOR = 4.90, 95% CI 1.72–13.98), not seeking nutrition content online (aOR = 5.32, 95% CI 2.03–13.96), low self-esteem (aOR = 7.88, 95% CI 2.19–28.37), and extreme body concern (aOR = 142.3, 95% CI 16.5–1228.7). Age group and BMI were not significant predictors. The final model demonstrated good fit (Hosmer–Lemeshow *p* = 0.41; Nagelkerke R^2^ = 0.39).

## 4. Discussion

The current study investigated the relationship between SM use, psychosocial factors, and the risk of EDs among preadolescent and adolescents enrolled in middle and high school. The prevalence of adolescents at risk of EDs was high (45.2%) in a young population, underscoring the relevance of this problem as a growing public health concern.

One of the most consistent findings was the strong association between prolonged daily SM use and risk of EDs. Adolescents who reported spending more than three hours per day on SM were over five times more likely to present with risk factors related to development of EDs compared with those with lower levels of use. These results are in sync with previous reports identifying excessive screen time as a risk marker for disordered eating behaviours [13]. While the current study identified significant associations between excessive use (>3 h/day) and risk of EDs, other authors have noted that frequency of visits may be an equally important predictor, suggesting that engagement patterns warrant closer scrutiny [32]. Additionally, most participants reported using social media primarily to access specific content, with video and image sharing being especially common on platforms such as Instagram and TikTok. These findings align with previous research indicating that image- and video-based social media are particularly popular among underage users [16,17,18,19]. Such platforms are widely used worldwide, and the theory of reasoned action supports the idea that, despite cultural differences, the underlying motivations for adoption, whether for personal benefit or social belonging, remain influential in shaping social media use [33].

It was confirmed that those who consume content to learn about nutrition (57.4%) or follow physical exercise routines (57.7%) are those who are most at risk of suffering from EDs, with *p*-values of 0.019 for nutrition and *p* < 0.001 for exercise. This hypothesis is supported by the previous literature, such as the study by Cohen et al. (2017) [34], where it was confirmed that following profiles dedicated to fitness and health is related to a greater idealization of thinness. Similar associations have been reported in earlier work, where following health and fitness accounts was linked to greater internalization of thinness ideals and body dissatisfaction [27]. Broader reviews also support this relationship, confirming that appearance-focused SM content increases vulnerability to body image concerns and EDs symptoms [35,36,37]. Exposure to diet- and exercise-related content on SM has been linked to thin-ideal internalization and body dissatisfaction, which may explain the observation that adolescents who used such content were at greater risk of EDs symptoms [36].

Although our results indicate that adolescents who consume SM content related to nutrition and physical exercise were more likely to be at risk of EDs, we did not explore whether the amount of time specifically dedicated to this type of content differed from other types of content. This distinction could help to clarify whether risk of EDs is linked to a more obsessive engagement with such content rather than its educational use. In this sense, a previous qualitative analysis identified how adolescents with a diagnosis of ED tend to use social media for content, such as diet, physical activity, appearance, and pro-ANA, focusing on the analysis of the content rather than the time of consumption [38].

Contrary to expectations, age was not significantly associated with ED risk factors, making it impossible to confirm one of the initial hypotheses. Although the highest prevalence was observed among younger adolescents (12–13 years, 48.7%), the differences were not statistically significant. These findings diverge from earlier work [17] in which age was identified as a risk factor, suggesting that the distribution of the present sample may have limited the detection of significant effects. A higher prevalence of risk of EDs was identified among females (53.9% vs. 30.8% in males), with girls more than twice as likely as boys to present contributors to EDs. These results align with longstanding evidence that adolescent girls are more vulnerable to body dissatisfaction and disordered eating [39,40]. These gender differences have been attributed to sociocultural pressures and exposure to unrealistic thin-ideal standards, which disproportionately affect adolescent girls [39].

BMI was not a significant predictor of EDs in this cohort, likely due to the predominance of participants within the healthy weight category (92%). Previous studies have shown stronger associations between higher BMI, body dissatisfaction, and an unhealthy relationship with food, suggesting that the small number of underweight and overweight/obese participants in the current sample may have precluded detection of such differences. Similarly, the age of onset of SM use was not statistically associated with EDs, although earlier initiation (<10 years) showed a descriptive trend toward higher vulnerability. A longitudinal study [40] has demonstrated that early exposure to SM amplifies the risk of disordered eating over time, indicating that cross-sectional designs may underestimate this effect.

Psychological variables were the strongest determinants of EDs. Low self-esteem increased the odds of EDs almost tenfold, while extreme body dissatisfaction was almost universally associated with high susceptibility to EDs. The strong correlation observed between self-esteem and body image supports previous findings that self-esteem mediates the relationship between emotional functioning and disordered eating [41,42]. Prior work also confirms that low self-esteem increases the likelihood of EDs during adolescence [43] and that self-esteem is inversely related to body dissatisfaction [44]. These findings emphasize the relevance of addressing psychosocial factors in preventive and intervention strategies.

Problematic use of SM was also strongly associated with adverse outcomes. Adolescents who spent more than three hours daily online were eighteen times more likely to present with social media disorder. In addition, those who reported using social media to escape negative feelings or failing to reduce use despite attempts were at significantly higher risk. These results are in line with previous research showing that maladaptive patterns of social media engagement are linked to addictive behaviours and negative personality development [45,46]. Recent systematic reviews further confirm that problematic social media use is associated with increased risks of depression, anxiety, and body dissatisfaction, which in turn intersect with disordered eating [47,48].

Overall, these findings underscore the need to reinforce nutritional and physical education in public schools. Such programmes should be delivered not only by subject-matter experts, such as healthcare providers, but also by professionals with pedagogical training, ensuring that adolescents receive accurate, age-appropriate, and engaging information that can counteract misleading or harmful content encountered online.

At the end, the findings highlight the complex interplay between digital behaviours, psychological well-being, and disordered eating in adolescence. SM acts both as a direct risk factor, through exposure time and type of content, and as an indirect risk factor by amplifying body dissatisfaction and lowering self-esteem. Although BMI and age did not emerge as significant predictors, gender differences and psychosocial variables played a particularly strong role. These results underscore the importance of school- and community-based interventions to promote media literacy, reduce appearance-based social comparisons, and strengthen self-esteem as a protective factor. Future research should expand to longitudinal designs, allowing for assessment of causal relationships and platform-specific effects, particularly given the growing influence of TikTok and Instagram on adolescent body image and eating behaviours.

### 4.1. Limitations

This study presents several limitations that must be considered when interpreting the findings. The primary limitation lies in the sample, as data were collected from a single school. Although significant associations were identified, the restricted scope limits the generalizability of the results. Expanding future samples to include multiple schools and diverse regions would strengthen representativeness and external validity.

Another limitation is the omission of sociodemographic and psychosocial variables such as cultural background, socioeconomic status, family environment, personality traits, and parental attitudes toward food. These factors may play a crucial role in shaping SM use, body image perception, and vulnerability to eating disorders. Additionally, while this study examined the relationship between SM use and disordered eating, no significant associations were observed with specific platforms. This may be due to the homogeneity of usage patterns within the sample, as most participants reported similar engagement with WhatsApp, Instagram, and TikTok, limiting variability for meaningful comparison. Future studies should therefore differentiate between platforms, levels of engagement, and types of content consumed, as these may exert distinct influences on risk.

An additional limitation worth noting is the reliance on self-report measures, which are inherently subject to potential biases such as social desirability, recall inaccuracies, and individual differences in interpretation of survey items. Although these tools provide valuable insights into participants’ perceptions and experiences, the possibility of over- or underreporting should be considered when interpreting the findings. Moreover, the study did not separately assess the time dedicated to different types of social media content (e.g., nutrition or exercise), which limits the ability to determine whether the observed risk is linked to the nature of the content, the intensity of exposure, or a combination of both.

### 4.2. Future Research

Future research could relate the variables included in this study with other proposals in other investigations that may influence how participants perceive themselves, such as those mentioned above in the limitations: cultural level, social environment together with personality variables, and parents’ attitudes towards food. It could also evaluate the effectiveness of various interventions designed to improve self-esteem and body self-perception in different populations. This comprehensive and multifaceted approach is essential to effectively address the growing problem of eating disorders in our society. Another line of research could be to conduct a comparative study between different cultures to verify whether these factors influence the relationship between the use of social networks and the risk of developing EDs.

## 5. Conclusions

In conclusion, the findings of this study highlight the strong association between self-esteem, body self-perception, and the risk of developing EDs within the context of SM use. Low self-esteem and negative body image emerged as key predisposing factors that increase vulnerability to unhealthy relationships with food. Adolescents who experience persistent dissatisfaction with their bodies and weakened self-esteem are more likely to adopt harmful eating behaviours in pursuit of unrealistic, socially imposed beauty ideals; pressures that are often amplified by content on SM platforms.

The results also indicate that problematic use of SM, whether due to excessive time spent online or the type of content consumed, particularly when it is used as a primary source of information, further contributes to the risk of developing EDs among young people.

These findings underscore the urgent need for preventive strategies and interventions that simultaneously promote healthy self-esteem, foster positive body image, and encourage critical, conscious use of SM. Educational initiatives aimed at strengthening self-worth from an early age, promoting realistic representations of body diversity, and equipping adolescents with skills to navigate online content responsibly may be effective tools in reducing the risk of EDs.

In addition, our findings highlight the importance of integrating nutritional and physical education into the public education system, delivered by experts with both subject knowledge and pedagogical training, to provide adolescents with reliable tools to critically navigate SM content.

## Figures and Tables

**Table 1:** Baseline sociodemographic characteristics of the cohort.

Qualitative Variables	Frequency	95% CI
Sex		
Male	52	45.2% (36.1–54.3%)
Female	63	54.8% (45.7–63.9%)
Total	115	100%
Academic year		
1st ESO	29	25.2% (17.3–33.1%)
2nd ESO	30	26.1% (18.1–34.1%)
3rd ESO	31	27.0% (18.9–35.1%)
4th ESO	25	21.7% (14.2–29.2%)
Total	115	100%
Quantitative Variables	Mean	Standard Deviation (SD)
Age	13.85	1.41

**Table 6:** Prevalence of risk of ED among adolescents (EAT-26).

Variable	Rate	95% CI
Eating Disorder (EAT-26)		
At risk of ED (≥11)	52/115	45.2%
Not at risk of ED (<11)	63/115	54.7%
Total	115	100%

**Table 7:** Factors associated with risk of ED: univariate analysis.

Variable	Without Risk	With Risk	OR	95% CI	*p*
Daily hours dedicated to social media?					
0–1 h	18	12	Ref		0.3637
1–3 h	37	16	1.54	(0.60–3.93)	
>3 h	9	21	5.54	(2.03–14.33)	<0.001
Social media content					
Exercise					
No	35	16	2.48	(1.15–5.359)	0.019
Yes	30	34	Ref		
Nutrition					
No	39	10	6.00	(2.56–14.07)	<0.001
Yes	26	40	Ref		
Age					
12–13 y/o	20	19	1.58	(0.33–7.56)	
14–15 y/o	40	28	1.17	(0.26–5.28)	0.705
16–17 y/o	5	3	Ref		
Gender					
Male	36	16	Ref		
Female	29	34	2.64	(1.22–5.69)	0.012
BMI					
Underweight/ Normal weight	63	46	Ref		
Overweight/Obesity	2	4	2.60	(0.45–14.9)	1.2443
Age of onset of social media					
<10 y/o	6	3	Ref		
10–12 y/o	30	23	1.53	(0.35–6.79)	
12–16 y/o	28	24	1.71	(0.39–7.60)	0.730
Did not use	1	0			
Self-esteem					
Low self-esteem (24–34)	9	31	9.64	(2.73–34.08)	<0.001
Medium self-esteem (24–34)	36	20	1.55	(0.49–4.95)	0.564
High self-esteem (35–40)	14	5	Ref		
Body image					
No concern	51	6	8.50	(2.65–27.23)	<0.001
Mild concern	12	12	55.2	(9.97–306.17)	<0.001
Moderate concern	2	13	5	(18.23–1430.97)	<0.001
Extreme concern	0	19	161.50		

**Table 8:** Predictors of SMD.

Variable	Without SM Disorder	With SM Disorder	OR	95% CI	*p*
Daily hours dedicated to social media?					
0–1 h	22	8	Ref		
1–3 h	46	7	2.39	(0.77–7.43)	
>3 h	8	22	18.07	(5.81–56.20)	<0.001
Age of onset of social media					
<10 y/o	5	4	2.03	(0.48–8.59)	
10–12 y/o	38	15	Ref		
12–16 y/o	33	19	1.46	(80.64–3.32)	0.05
Have you tried to spend less time on social media but failed?					
No	49	9	Ref		
Yes	28	29	5.64	(2.33–13.60)	<0.001
Do you use social media to escape from negative feelings?					
No	44	4	Ref		
Yes	33	34	11.33	(3.66–35.09)	<0.001

**Table 9:** Association between self-esteem and body image perception in relation to risk of ED.

Variable	Low Self-Esteem	Medium Self-Esteem	High Self-Esteem	*p*
Body self-perception				
No concern	5	18	39	<0.001
Mild concern	12	13	3	<0.001
Moderate concern	5	9	4	<0.001
Extreme concern	18	6	0	<0.001

**Table 2:** Baseline anthropometric characteristics of the cohort.

Quantitative Variables	Mean	SD
Weight	53.2	10.7
Height	161.4	17.0
BMI	20.04	2.55
Qualitative Variables	Frequency	95% CI
BMI		
Underweight	3	2.6% (1.8–3.4%)
Healthy Weight	106	92.2% (87.3–97.1%)
Overweight I	1	0.9% (0.8–2.5%)
Overweight II	1	0.9% (0.8–2.5%)
Obesity	4	3.5% (2.6–4.3%)
Total	115	100%

**Table 3:** Baseline SM use characteristics of the cohort.

Variable	Frequency	95% CI
Do you usually use SM in your daily life?		
Yes	111	96.5% (93.1–99.9%)
No	4	3.5% (2.6–4.3%)
Total	115	100%
At approximately what age did you start using social media?		
<10 y/o	9	7.8% (7.0–8.6%)
10–12 y/o	53	46.1% (37.0–55.2%)
12–16 y/o	52	45.2% (36.1–54.3%)
>16 y/o	1	0.9% (0.8–2.5%)
Total	115	100%
How much time do you spend in total in your day-to-day life using social media?		
<30 min/day	5	4.3% (1.0–8.0%)
30 min–1 h/day	25	21.7% (14.2–29.2%)
1–3 h/day	53	46.1% (37.0–55.2%)
>3 h/day	30	26.1% (18.1–34.1%)
I do not use social media	1	1.7% (1.0–2.4%)
Total	115	100%
Do you feel that the content you view on social media negatively affects you?		
Yes	42	36.5% (27.7–45.3%)
No	73	63.5% (54.7–72.3%)
Total	115	100%
Do you use social media to follow physical exercise routines, in addition to other content?		
Yes	64	55.7% (46.6–64.8%)
No	51	44.3% (35.2–53.4%)
Total	115	100%
Do you use social media to learn about nutrition and search for recipes, in addition to other content?		
Yes	66	57.4% (48.4–66.4%)
No	49	42.6% (33.6–51.6%)
Total	115	100%
Social Media Disorder Scale		
Social media disorder	38	33.0% (24.4–41.6%)
No social media disorder	77	67.0% (58.4–75.6%)
Total	115	100%

**Table 4:** Baseline lifestyle characteristics of the cohort.

Variables	Frequency	95% CI
Do you workout?		
Yes	101	87.8% (81.8–93.8%)
No	13	11.3% (5.5–17.1%)
Sometimes	1	0.9% (0.8–2.5%)
Total	115	100%
Why do you workout?		
Health/Fun	55	47.8% (38.7–56.9%)
Body dissatisfaction	18	15.7% (9.1–22.4%)
Both	42	36.5% (22.7–45.3%)
Total	115	100%

**Table 5:** Baseline psychosocial characteristics: self-esteem and body image.

Variable	Frequency	95% CI
Rosenberg Self-Esteem Test		
Low self-esteem (10–24)	40	34.7% (26.0–43.4%)
Medium self-esteem (24–34)	56	48.7% (39.6–57.8%)
High self-esteem (35–40)	19	16.5% (9.7–23.3%)
Total	115	100%
Body Shape Questionnaire-14		
No concern (14–33)	57	49.5% (40.4–58.6%)
Mild concern (34–45)	23	20% (12.7–27.3%)
Moderate concern (46–60)	16	13.9% (7.6–20.2%)
Extreme concern (61–84)	19	16.5% (9.7–23.3%)
Total	115	100%

## Data Availability

The data are available under contact with the corresponding author.

## Abbreviations

The following abbreviations are used in this manuscript:

ANOVA	Analysis of Variance
BMI	Body Mass Index
BSQ	Body Shape Questionnaire
CI	Confidence Interval
DSM-5	Diagnostic and Statistical Manual of Mental Disorders, Fifth Edition
EAT-26	Eating Attitudes Test-26
ED/EDs	Eating Disorder(s)
ESO	Educación Secundaria Obligatoria (Spanish Compulsory Secondary Education)
ICTs	Information and Communication Technologies
OR	Odds Ratio
SD	Standard Deviation
SM	Social Media
SMDS	Social Media Disorder Scale
WHO	World Health Organization
